# A case with congenital absence of the left atrial appendage

**DOI:** 10.1002/joa3.70120

**Published:** 2025-06-23

**Authors:** Kosuke Muto, Naomichi Tanaka, Hitoshi Mori, Yoshifumi Ikeda, Ritsushi Kato

**Affiliations:** ^1^ Department of Cardiology Saitama Medical University, International Medical Center Saitama Japan

**Keywords:** left atrial appendage

## Abstract

Congenital absence of the left atrial appendage (LAA) is an extremely rare anatomical variation with important implications for the management of atrial fibrillation (AF). Additionally, atrial natriuretic peptide (ANP) production may be compensated by the right atrial appendage (RAA) in cases of congenital LAA deficiency.

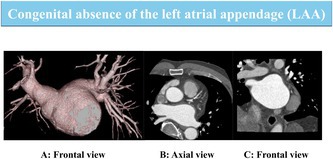

Congenital absence of the left atrial appendage (LAA) is an extremely rare anatomical anomaly, with very few reports in the medical literature. The LAA plays a crucial role in thrombus formation, which can potentially lead to cerebrovascular events. Therefore, in patients with atrial fibrillation (AF) undergoing cardioversion or ablation therapy, it is essential to confirm the absence of thrombi in the LAA.

However, there is no established guideline on how to manage anticoagulation therapy after ablation in patients diagnosed with congenital LAA absence. The decision must be made based on an individualized risk assessment for each patient.^1^

Additionally, the LAA is involved in the production of atrial natriuretic peptide (ANP). While there have been reports on ANP levels following percutaneous LAA closure and epicardial LAA closure (LAAC), no studies have documented ANP levels in patients with congenital absence of the LAA.^2^, ^3^

A 51‐year‐old man with a history of hypertension and hyperuricemia was referred to our hospital for further evaluation and treatment after AF was detected during a routine health check‐up, following his longstanding awareness of palpitations (Figure 1). His CHA_2_DS_2_VASc score was 1, and his HAS‐BLED score was also 1.

**FIGURE 1:**
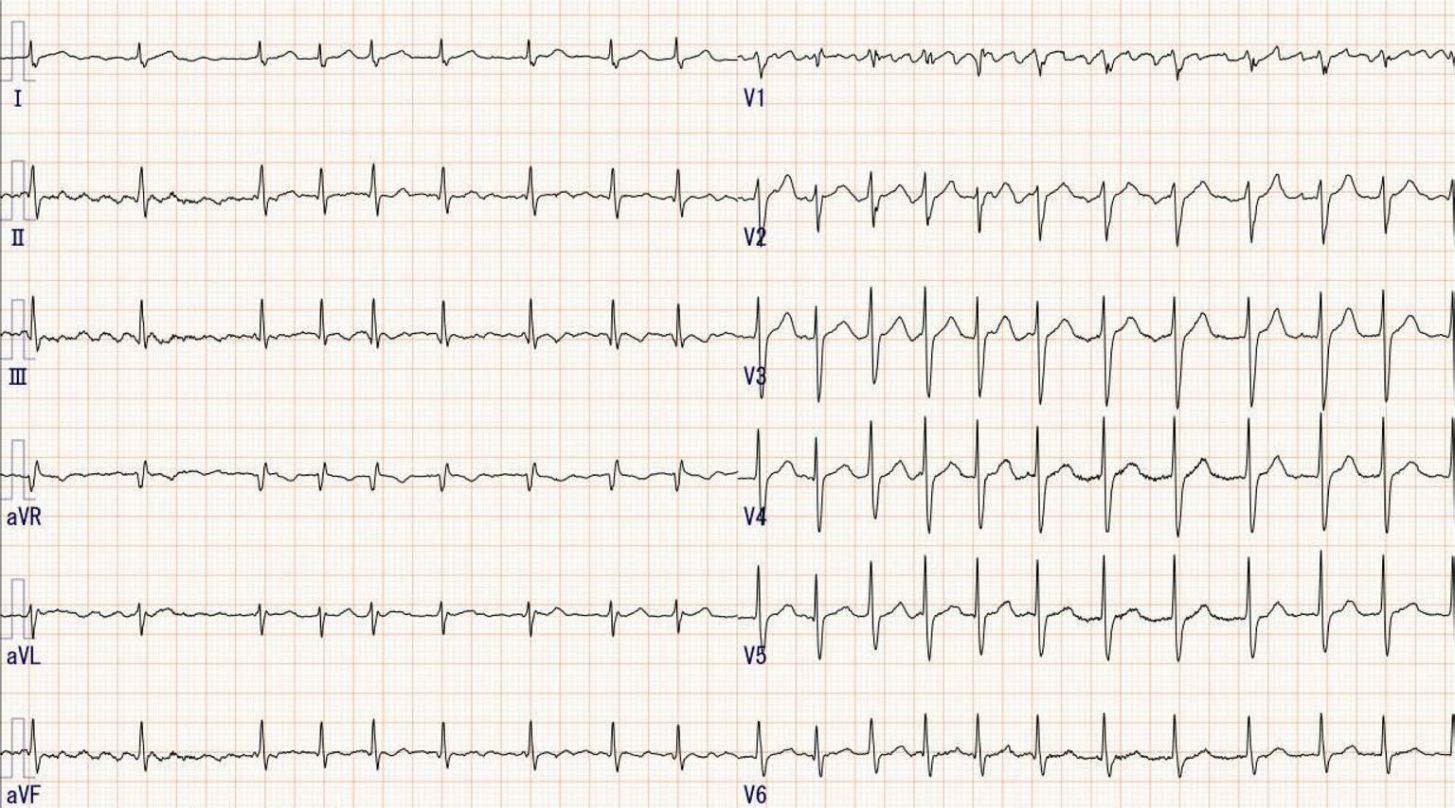
Electrocardiogram (ECG) at admission.

A contrast‐enhanced CT scan was performed preoperatively to assess for thrombus before ablation. The scan revealed no significant pulmonary vein anomalies, and all pulmonary veins were perfusing into the left atrium. Additionally, congenital absence of the LAA was noted (Figure 2). As the patient had no history of previous cardiac surgery, the absence of the LAA was considered congenital.

**FIGURE 2:**
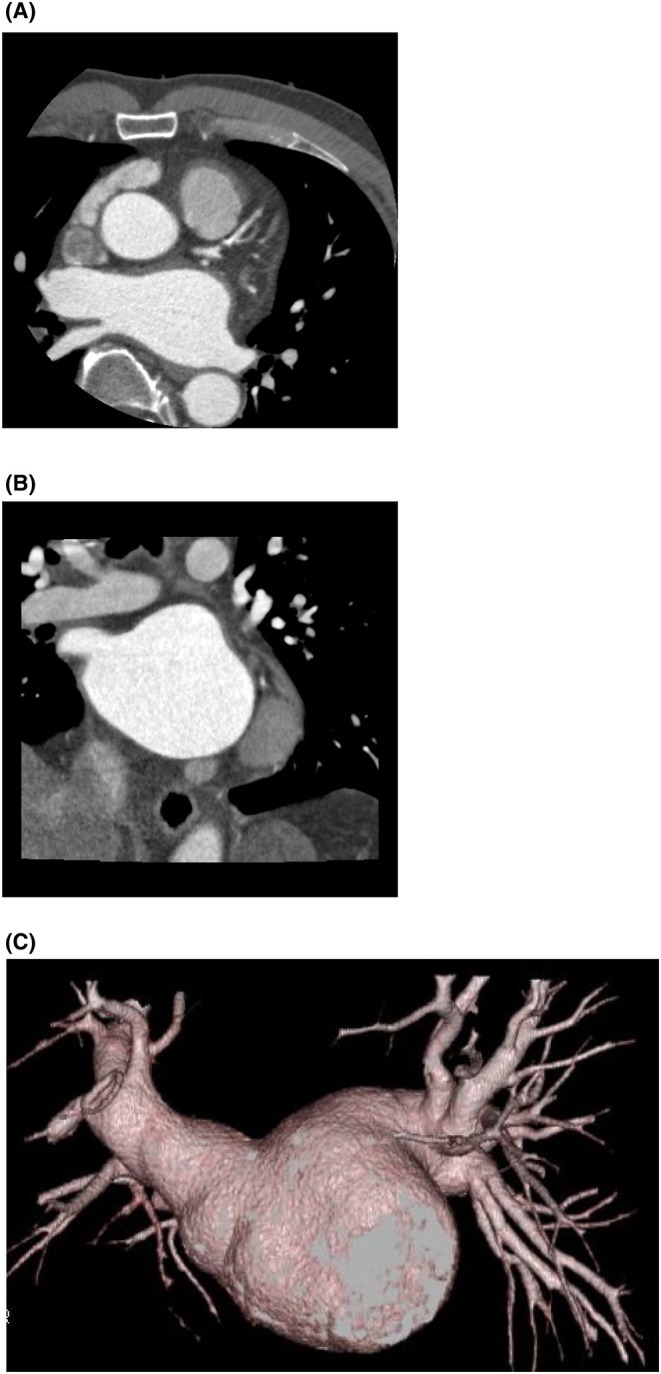
Preoperative contrast‐enhanced CT for ablation revealed congenital absence of the left atrial appendage (LAA). (A: Frontal view, B: Left lateral view).

Ablation was performed using the CRYO system, isolating all four pulmonary veins along with the posterior wall. LAA pacing was omitted with reference to the preprocedural CT images, and the procedure was completed successfully. During the catheter procedure, no anatomical structure consistent with the left atrial appendage was identified. Considering the risk of atrial fibrillation recurrence, dabigatran etexilate mesylate (300 mg) was prescribed for 3 months postoperatively. The patient was discharged home in stable condition.

The LAA develops from the left wall of the primary atrium during fetal life and functions similarly to the left atrium. In adults, it is thought to act as a decompression chamber when left atrial pressure increases, such as during left ventricular systole or volume overload. Additionally, the LAA contributes to the reservoir and contractile functions of the left atrium and plays a significant role in the high production of ANP.^4^

The LAA plays a potential role in AF and thromboembolism. In patients with nonvalvular AF, approximately 90% of thrombi have been reported to form within the LAA. Additionally, in about 27% of patients undergoing repeat ablation, the LAA has been identified as a trigger site for AF.^2^

In the present case, LAA absence was not detected on transthoracic echocardiography (TTE) but was identified through contrast‐enhanced CT scanning. Previous reports have also documented cases where transesophageal echocardiography (TEE) confirmed LAA absence.^1^ Given these findings, preoperative imaging with contrast‐enhanced CT or TEE is crucial to assess LAA morphology before AF ablation.

There are currently no established guidelines for anticoagulation therapy after AF ablation in patients with congenital absence of the LAA.^1^ For antithrombotic and anticoagulation therapy after percutaneous LAA closure, it has been recommended that: for the first 45 days postdevice implantation, patients should receive a combination of anticoagulants and antiplatelet agents. Up to 6 months postprocedure, dual antiplatelet therapy (DAPT) should be continued. After 6 months, a single antiplatelet agent (SAPT) should be administered. On the other hand, for thoracoscopic LAA closure, some reports indicate that anticoagulation therapy could be discontinued postoperatively without increasing the risk of stroke.^2^ However, because of limited long‐term data on stroke risk in such patients, caution is required at this stage. Until more definitive evidence becomes available, a cautious and reasonable approach would be to continue anticoagulation therapy while gradually reducing its intensity.

In this case, considering the potential for acute‐phase recurrence of AF postoperatively, the patient was discharged home with a plan to continue anticoagulation therapy for 3 months. With a CHA_2_DS_2_‐VASc score of 1 and a HAS‐BLED score of 1, the patient had a low risk of both stroke and bleeding. If AF does not recur, the decision regarding the continuation or discontinuation of anticoagulation therapy should be carefully discussed with the patient and made with caution.

The LAA is also involved in the production of ANP. While reports exist on ANP levels following transcatheter LAA closure and percutaneous epicardial LAA closure, there have been no reports on ANP in cases of congenital LAA deficiency.^2^, ^3^ Regarding ANP levels after transcatheter LAA closure, ANP levels increased immediately after the procedure but showed a significant decrease compared to baseline levels before discharge.^2^ Similarly, in percutaneous epicardial LAA closure, ANP levels significantly increased 24 h postprocedure but significantly decreased at 7 days, 1 and 3 months postprocedure. However, at 12 and 24 months, there was no change compared to baseline.^3^ Since ANP is also produced in the right atrial appendage (RAA), it is possible that, over time, the RAA compensates for the loss of ANP production after LAA closure. In the present case, the ANP level was within the normal range at 29.8 pg/mL, suggesting that even in congenital LAA deficiency, ANP production may be compensated by the RAA.

In conclusion, congenital absence of the LAA is a very rare anatomical variation that has significant implications for the management of AF. When considering anticoagulation therapy, a careful selection should be made based on comprehensive imaging diagnostics and individual patient risk assessment. Additionally, while the LAA is involved in ANP production, in cases of congenital LAA deficiency, ANP may be compensated by production in the RAA.

## CONFLICT OF INTEREST STATEMENT

The authors report no conflicts of interest.

## ETHICS STATEMENT

Informed consents were obtained from the patients to publish the case report.
